# White blood cell counts can predict 4-year cardiovascular disease risk in patients with stable coronary heart disease: a prospective cohort study

**DOI:** 10.3389/fcvm.2024.1358378

**Published:** 2024-09-26

**Authors:** Wencai Jiang, Gang Huang, Jinfeng Du, Hanxuan Yang, Shiheng Zhou, Dayin Dai, Kai Tang, Lingxiao Fang, Xiao Wang, Xuejun Deng

**Affiliations:** ^1^ Department of Cardiology, Suining Central Hospital, Suining, Sichuan, China; ^2^ Department of Hematology, Chongqing Emergency Medical Center, Chongqing, China

**Keywords:** white blood cell (WBC) count, risk factors, cardiovascular disease, cardiovascular events, stable coronary heart disease

## Abstract

**Background:**

The prevalence of cardiovascular disease has increased sharply in the Asian population, and evaluation of the risk of cardiovascular events with stable coronary heart disease remains challenging. The role of white blood cell (WBC) count in assisting clinical decision-making in this setting is still unclear.

**Objectives:**

This study sought to evaluate the prognostic meaning of WBC count among patients with stable coronary heart disease.

**Methods:**

This study included Asian participants (*n* = 1,933) from the prospective STABILITY (Stabilization of Atherosclerotic Plaque by Initiation of Darapladib Therapy) trial, which involved 15,828 patients with stable coronary heart disease with 3–5 years of follow-up on optimal secondary preventive treatment. WBC count was measured at baseline. Associations between WBC count and cardiovascular outcomes were evaluated by Cox regression analyses with multivariable adjustments. Hematologic emergencies in patients may introduce potential bias.

**Results:**

In the lower WBC count quartiles, patients had lower-risk clinical profiles. Higher WBC counts were associated with greater event probabilities for cardiovascular death, major cardiovascular events, or all-cause death. In Cox regression models, WBC counts were an independent predictor of major adverse cardiovascular events (OR = 2.445, 95% CI 1.427–4.190, *P* = 0.001) for the primary outcomes. For the secondary outcomes, including the composite of all-cause death, cardiovascular death, myocardial infarction, stroke, and hospitalization for heart failure, WBC counts were significantly predictive of events with similar magnitude (OR = 1.716, 95% CI 1.169–2.521, *P* = 0.006).

**Conclusions:**

In patients with stable coronary heart disease, higher WBC counts were associated with a heightened risk for the primary or secondary outcomes.

**Registration:**

https://clinicaltrials.gov/; Unique identifier NCT00799903.

## Introduction

Cardiovascular diseases are increasing worldwide. In Asia, the aging population has led to a particularly significant increase in the prevalence, morbidity, and mortality of cardiovascular diseases (1). This poses a huge medical burden on the families and society in this area. With the maturity and development of percutaneous coronary intervention (PCI), more and more people are surviving with these diseases (2). Increased attention to promoting ideal cardiovascular health in patients with stable coronary heart disease is necessary (3). However, evaluation of the prognosis of patients with stable coronary heart disease remains challenging. Although coronary computed tomography angiography (CTA) and coronary angiography are useful, these imaging modalities have limitations, including availability, cost, the need for specialized interpretation, and exposure to ionizing radiation (4). It is time to implement feasible and affordable strategies for the prevention and control of stable coronary heart disease and to monitor results (5).

The role of white blood cell (WBC) count in predicting the risk of coronary heart disease in a normal population has been confirmed (6–8). However, compared to other regions, there are differences in eating habits, risk factors, and gene frequencies among Asian people (9, 10). Most of the existing literature focuses on the relationship between WBC count and acute myocardial infarction (MI) but neglects the role of WBC count in managing patients with coronary heart disease. The role of WBC count in predicting the risk of recurrent cardiovascular events in patients with stable coronary heart disease remains uncertain. Our study is a *post-hoc* analysis of the prospective STABILITY (Stabilization of Atherosclerotic Plaque by Initiation of Darapladib Therapy) trial, which is a large-scale global trial with a large amount of data on patients with stable coronary heart disease from multiple centers in Asia. The aim of the current study was to explore the associations between WBC count and clinical outcomes in patients with stable coronary heart disease, with the goal of augmenting the ability to triage patients properly with stable coronary heart disease and assist in clinical interpretation and decision-making. In addition, we aimed to better understand the potential mechanisms behind the associations.

## Methods

The research data of this article can be available from https://search.vivli.org/doiLanding/studies/00000708/isLanding upon an appropriate request.

### Population

Our study data were obtained from the prospective STABILITY trial, a randomized placebo-controlled study evaluating the effects of a lipoprotein-associated phospholipase A2 inhibitor, darapladib. It included 15,828 patients with stable coronary heart disease from 39 countries (11), with a follow-up of 3–5 (median, 3.7) years on optimal secondary preventive treatment. We analyzed the data on the Asian population (*n* = 1933), including China (*n* = 178, 25.9%), Japan (*n* = 110, 34.6%), the Republic of Korea (*n* = 107, 21.3%), the Philippines (*n* = 20, 9.1%), and Thailand (*n* = 43, 20.8%). The trial design, inclusion and exclusion criteria, baseline characteristics, and endpoint events have been described previously (12, 13). The trial was performed in accordance with the Declaration of Helsinki. In each country, the study was approved by national regulatory authorities and by local ethics committees or institutional review boards, according to local regulations. All participants provided written informed consent for their participation. It was funded by GlaxoSmithKline and is registered at ClinicalTrials.gov (unique identifier: NCT00799903; https://clinicaltrials.gov/ct2/show/NCT00799903).

### Grouping and outcomes

WBC counts were measured at baseline in the Asian population (*n* = 1,933). The study population was divided into four groups according to the quartiles of WBC counts: WBC < 5.3 GI/L, WBC < 6.3 GI/L, WBC < 7.5 GI/L, and WBC ≥ 7.5 GI/L. Venous blood samples from all participants were obtained in the morning after at least 8 h of fasting. The method of laboratory examination has been described in previously published studies (14, 15). Definitions of all endpoints were prespecified and distinctly represented before (12). These were adjudicated by an independent clinical events committee. The primary outcomes, defined as major adverse cardiovascular events (MACEs), included cardiovascular death, myocardial infarction, and stroke. The secondary outcomes included all-cause death, cardiovascular death, myocardial infarction, stroke, and hospitalization for heart failure. We analyzed cardiovascular event outcomes over about 4 years in participants with different white blood cell counts. All laboratory tests were performed at central laboratories (Quest Diagnostics Clinical Laboratories) to ensure the reliability of the WBC count.

### Statistical analysis

Categorical variables were represented by percentages, which will be compared using the chi-square test. Continuous variables were compared using Kruskal–Wallis non-parametric tests. A Cox proportional hazards model was used to analyze and compare the incidence of cardiovascular events of patients with chronic coronary heart disease with different WBC counts. In the multivariable model, we adjusted for the following factors: age, sex, country, BMI, waist/hip ratio, systolic blood pressure, diastolic blood pressure, hemoglobin, hematocrit, neutrophils, platelets, blood lipids, high-sensitivity C-reactive protein (hsCRP), creatinine, estimated glomerular filtration rate (eGFR), urea/blood urea nitrogen (BUN), glucose, liver function, prior MI, multivessel coronary heart disease, prior coronary artery bypass grafting (CABG), diabetes, hypertension, chronic obstructive pulmonary disease (COPD), clopidogrel bisulfate, aspirin, angiotensin-converting enzyme inhibitors/angiotensin receptor blockers (ACEis/ARBs), statins, β-receptor blockers, and smoking status. We used multiple imputations to deal with missing data. All statistical analyses were performed using SPSS 26.0 (IBM, New York City, NY, USA). If *P* < 0.05, the difference was statistically significant.

## Results

### Baseline characteristics of patients in different white blood cell count groups

**Figure 1:**
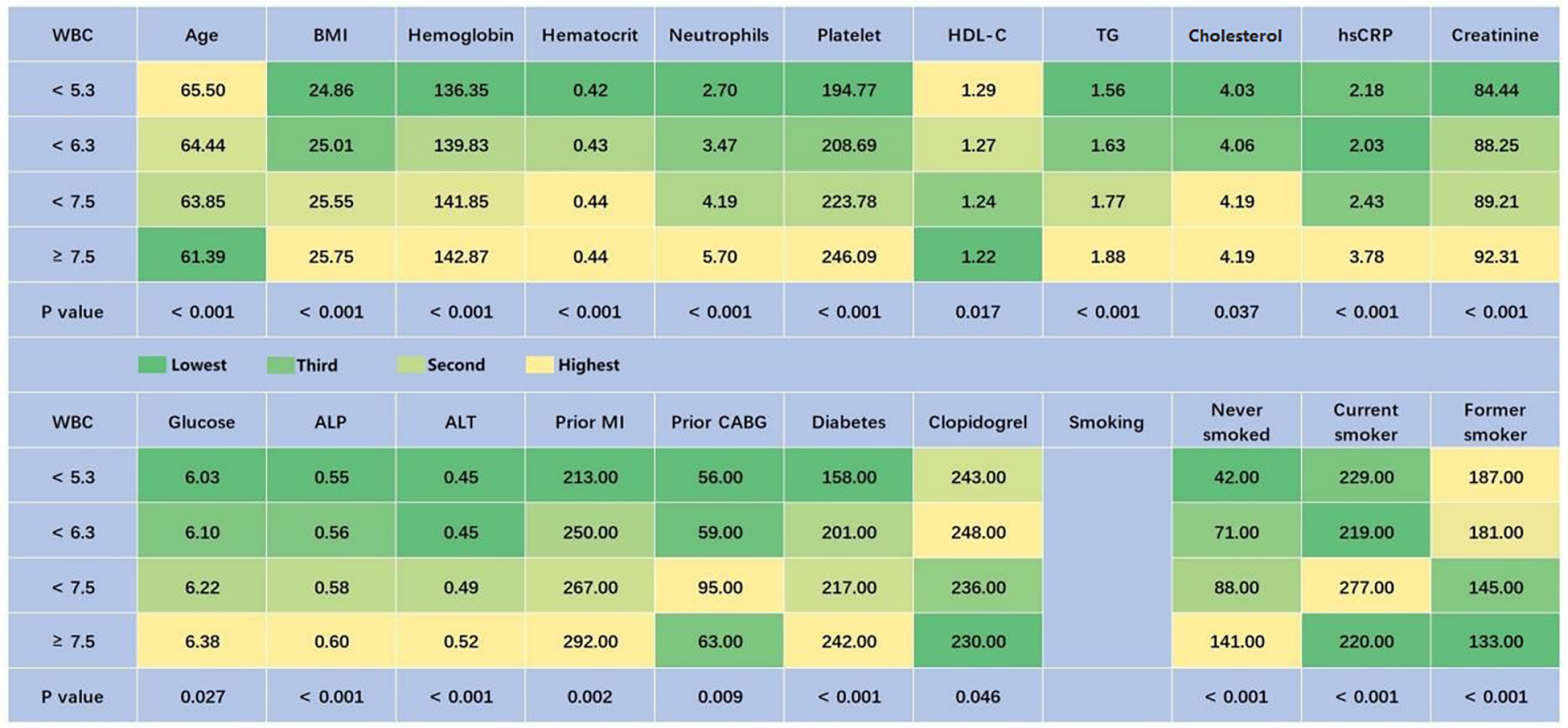
Baseline characteristics of patients with different quartiles of WBC (predictors with *P* < 0.05, excepting gender and country). Green represents the lowest value, and yellow represents the highest value. ALP, alkaline phosphatase; ALT, alanine aminotransferase; BMI, body mass index; CABG, coronary artery bypass grafting; HDL-C, high-density lipoprotein cholesterol; hsCRP, high-sensitivity C-reactive protein; MI, myocardial infarction; TG, triglyceride; WBC, white blood cell.

The average age of the study population (*n* = 1,933) was 63.75 ± 9.40 years. Among them, 408 were women, accounting for 21.1% of the population. In total, 458 patients had a WBC count of <5.3 GI/L, 471 had a WBC count of <6.3 GI/L, 510 had a WBC count of <7.5 GI/L, and 494 had a WBC count of ≥7.5 GI/L. Among patients with stable coronary heart disease, older age was associated with lower white blood cell counts. In the WBC count <5.3 GI/L group, the proportion of females was the highest (26.2%). Patients from different countries exhibited different WBC counts. The baseline characteristics and demographics are presented in Table 1. There were statistically significant differences in sex, country, BMI, hemoglobin, hematocrit, neutrophils, platelets, high-density lipoprotein cholesterol (HDL-C), triglycerides (TGs), cholesterol, hsCRP, creatinine, glucose, alkaline phosphatase (ALP), alanine aminotransferase (ALT), prior MI, prior CABG, diabetes, clopidogrel, and smoking status among patients with different WBC counts (*P* < 0.05). To facilitate observation, except for gender and country, we created Figure 1 to highlight predictors with obvious differences and expressed their numerical values with different color scales. In the WBC count <5.3 GI/L group, BMI, hemoglobin, hematocrit, neutrophils, platelets, TGs, cholesterol, hsCRP, creatinine, glucose, ALP, ALT, prior MI, prior CABG, diabetes, and the percentage of never smokers were the lowest, while age, HDL-C, and the percentage of former smokers were the highest.

**Table 1:** Baseline characteristics by WBC count quartiles.

Baseline characteristics	<5.3 GI/L *n* = 458	<6.3 GI/L *n* = 471	<7.5 GI/L *n* = 510	≥7.5 GI/L *n* = 494	*P*-value
Age (years)	65.50 ± 8.34	64.44 ± 9.23	63.85 ± 9.42	61.39 ± 10.00	<0.001
Female, *n* (%)	120 (26.2)	94 (20.0)	97 (19.0)	97 (19.6)	0.023
Country, *n* (%)					
China	178 (25.9)	170 (24.8)	172 (25.1)	166 (24.2)	<0.001
Japan	110 (34.6)	87 (27.4)	79 (24.8)	42 (13.2)	<0.001
Republic of Korea	107 (21.3)	132 (26.2)	141 (28.0)	123 (24.5)	<0.001
Philippines	20 (9.1)	40 (18.3)	66 (30.1)	93 (42.5)	<0.001
Thailand	43 (20.8)	42 (20.3)	52 (25.1)	70 (33.8)	<0.001
BMI, kg/m^2^	24.86 ± 3.07	25.01 ± 3.28	25.55 ± 3.36	25.75 ± 3.6	<0.001
Waist/hip ratio	0.93 ± 0.07	0.93 ± 0.07	0.93 ± 0.08	0.93 ± 0.07	0.497
SBP, mmHg	75.96 ± 11.13	77.45 ± 10.79	77.24 ± 11.69	77.32 ± 11.03	0.254
DBP, mmHg	130.67 ± 16.91	130.19 ± 16.41	131.08 ± 17	130.12 ± 16.32	0.757
Hemoglobin, g/L	136.35 ± 15.59	139.83 ± 14.32	141.85 ± 14.57	142.87 ± 16.46	<0.001
Hematocrit	0.42 ± 0.05	0.43 ± 0.04	0.44 ± 0.04	0.44 ± 0.05	<0.001
Neutrophils, 10^9^/L	2.7 ± 0.5	3.47 ± 0.52	4.19 ± 0.62	5.7 ± 1.44	<0.001
Platelets, 10^9^/L	194.77 ± 46.66	208.69 ± 50.51	223.78 ± 53.84	246.09 ± 64.83	<0.001
HDL-C, mmol/L	1.29 ± 0.36	1.27 ± 0.34	1.24 ± 0.31	1.22 ± 0.32	0.017
LDL-C, mmol/L	2.04 ± 0.7	2.06 ± 0.74	2.15 ± 0.84	2.13 ± 0.84	0.228
TGs, mmol/L	1.56 ± 0.96	1.63 ± 0.98	1.77 ± 0.93	1.88 ± 1.61	<0.001
cholesterol, mmol/L	4.03 ± 0.88	4.06 ± 0.83	4.19 ± 0.94	4.19 ± 0.94	0.037
hsCRP, mg/L	2.18 ± 3.79	2.03 ± 2.76	2.43 ± 3.8	3.78 ± 8.76	<0.001
Creatinine, µmol/L	84.44 ± 20.74	88.25 ± 21.87	89.21 ± 21.68	92.31 ± 26.23	<0.001
eGFR (CKD-EPI)	1.34 ± 0.32	1.32 ± 0.33	1.31 ± 0.31	1.31 ± 0.36	0.230
Urea/BUN	5.76 ± 1.66	5.89 ± 1.75	5.95 ± 1.82	6.09 ± 1.97	0.129
Glucose, mmol/L	6.03 ± 2.3	6.1 ± 1.72	6.22 ± 1.92	6.38 ± 2.15	0.027
Total bilirubin, mg/dl	0.6 ± 0.22	0.61 ± 0.21	0.59 ± 0.22	0.59 ± 0.23	0.259
ALP, µmol s^−1^/L	0.55 ± 0.15	0.56 ± 0.17	0.58 ± 0.19	0.6 ± 0.2	<0.001
ALT, nmol s^−1^/L	0.45 ± 0.31	0.45 ± 0.25	0.49 ± 0.29	0.52 ± 0.39	<0.001
AST, nmol s^−1^/L	0.47 ± 0.24	0.46 ± 0.25	0.47 ± 0.28	0.48 ± 0.26	0.750
Prior MI, *n* (%)	213 (46.5)	250 (53.1)	267 (52.4)	292 (59.1)	0.002
Multivessel CHD, *n* (%)	41 (9.0)	42 (8.9)	55 (10.8)	64 (13.0)	0.130
Prior CABG, *n* (%)	56 (12.2)	59 (12.5)	95 (18.6)	63 (12.8)	0.009
Diabetes, *n* (%)	158 (34.5)	201 (42.7)	217 (42.5)	242 (49.0)	<0.001
Hypertension, *n* (%)	341 (74.5)	339 (72.9)	372 (72.9)	373 (75.5)	0.606
COPD, *n* (%)	4 (0.9)	10 (2.1)	14 (2.7)	14 (2.8)	0.107
Clopidogrel, *n* (%)	243 (53.1)	248 (52.7)	236 (46.3)	230 (46.6)	0.046
Aspirin, *n* (%)	431 (94.1)	436 (92.6)	483 (94.7)	462 (93.5)	0.561
ACEi/ARB, *n* (%)	347 (75.8)	373 (79.2)	398 (78.0)	392 (79.4)	0.523
Statins, *n* (%)	441 (96.3)	460 (97.7)	499 (97.8)	474 (96.0)	0.210
β-receptor blockers, *n* (%)	198 (42.3)	195 (41.4)	229 (44.9)	221 (44.7)	0.668
Smoking status, *n* (%)					
Never smoked	42 (12.3)	71 (20.8)	88 (25.7)	141 (41.2)	<0.001
Current smoker	229 (24.2)	219 (23.2)	277 (29.3)	220 (23.3)	<0.001
Former smoker	187 (28.9)	181 (28.0)	145 (22.4)	133 (20.6)	<0.001

ACEi, angiotensin-converting enzyme inhibitor; ALP, alkaline phosphatase; ALT, alanine aminotransferase; ARB, angiotensin receptor blocker; AST, aspartate aminotransferase; BMI, body mass index; CABG, coronary artery bypass grafting; CDK-EPI, chronic kidney disease epidemiology collaboration; CHD, coronary heart disease; DBP, diastolic blood pressure; eGFR, estimated glomerular filtration rate; COPD, chronic obstructive pulmonary disease; HDL-C, high-density lipoprotein cholesterol; hsCRP, high-sensitivity C-reactive protein; LDL-C, low-density lipoprotein cholesterol; MI, myocardial infarction; PCI, percutaneous coronary intervention; SBP, systolic blood pressure; TG, triglyceride; WBC, white blood cell.

### WBC count and its association with clinical outcomes

By 4 years, 133 study participants experienced the primary outcomes of MACEs, whereas 214 participants experienced the secondary outcomes that included the composite of all-cause death, cardiovascular death, myocardial infarction, stroke, and hospitalization for heart failure. Patients who experienced secondary outcomes by 4 years had higher WBC counts at enrollment than those who did not (6.89 ± 1.75 GI/L vs. 6.50 ± 1.81 GI/L; *P* = 0.003). The associations between WBC counts and the risk of hospitalization for heart failure, stroke, MI, cardiovascular death, and all-cause death are presented in Table 2. By 4 years, the rates of all-cause death, MACEs, and cardiovascular death in patients with WBC count <5.3 GI/L were significantly lower than those with WBC count ≥5.3 GI/L, with *P*-values of 0.012, 0.028, and 0.033, respectively. The event probabilities for hospitalization for heart failure, stroke, or MI across WBC count quartiles were not significant.

**Table 2:** Clinical outcomes during follow-up by WBC counts.

Endpoint, *n* (%)	<5.3 GI/L *n* = 458	<6.3 GI/L *n* = 471	<7.5 GI/L *n* = 510	≥7.5 GI/L *n* = 494	*P*-value
Hospitalization for heart failure	9 (2.0)	6 (1.3)	12 (2.4)	11 (2.2)	0.628
Stroke	7 (1.5)	12 (2.5)	9 (1.8)	17 (3.4)	0.188
MI	7 (1.5)	15 (3.2)	18 (3.5)	18 (3.6)	0.199
Cardiovascular death	3 (0.7)	14 (3.0)	16 (3.1)	16 (3.2)	0.033
MACE	15 (3.3)	38 (8.1)	37 (7.3)	43 (8.7)	0.028
All-cause death	10 (2.2)	24 (5.1)	25 (4.9)	33 (6.7)	0.012

MACE, major cardiovascular event (cardiovascular death, MI, and stroke); MI, myocardial infarction.

**Figure 2:**
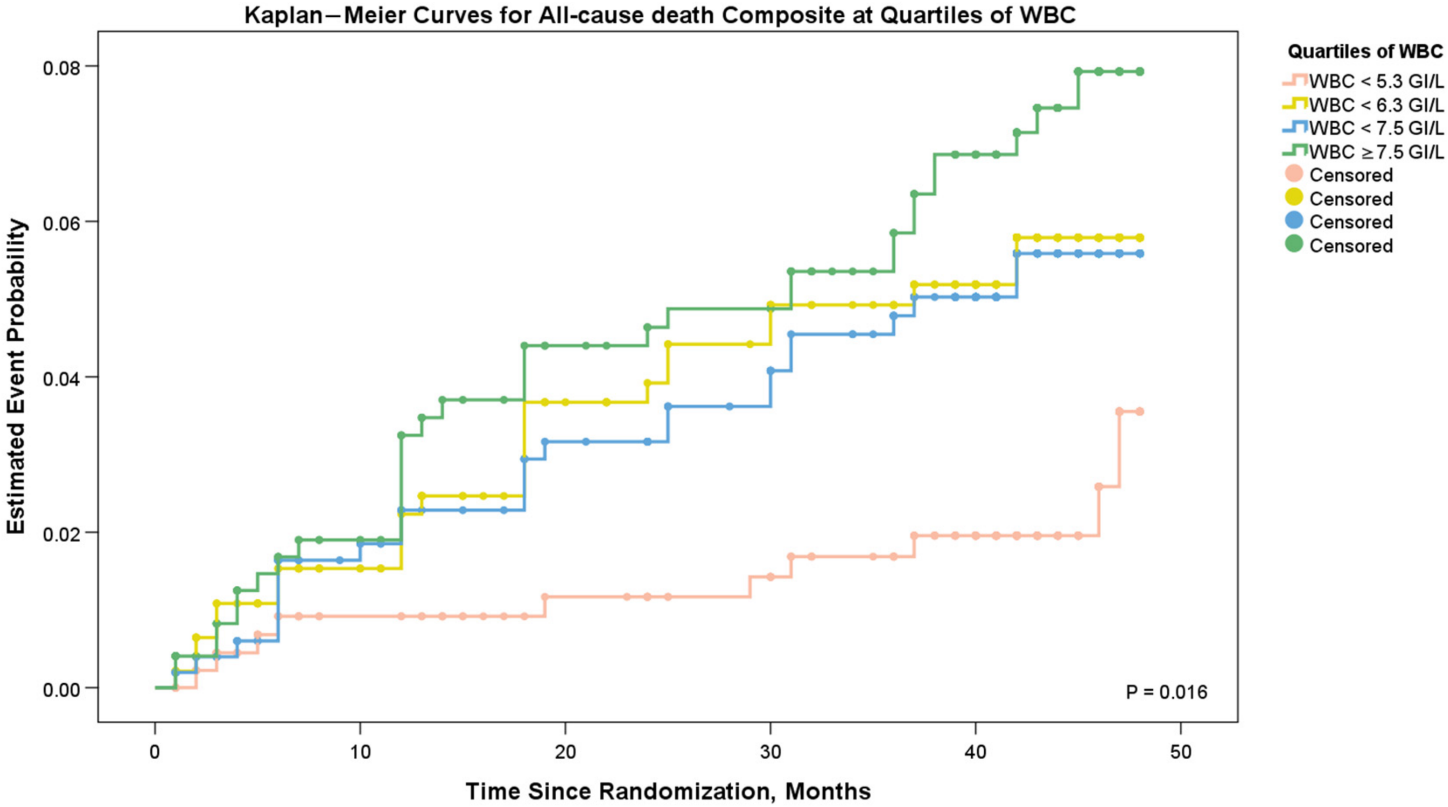
Cumulative hazard curves depicting time-to-events as a function of WBC quartiles at presentation.

We drew cumulative hazard curves, depicting time-to-events as a function of WBC quartiles at presentation (Figure 2). Kaplan–Meier curves suggest that the all-cause mortality in the WBC count <5.3 GI/L group was significantly lower than in the WBC count ≥5.3 GI/L groups. The log-rank test suggests that the difference in their estimated event probabilities was statistically significant (*P* = 0.016).

### Results of multivariate Cox proportional hazards models

**Figure 3:**
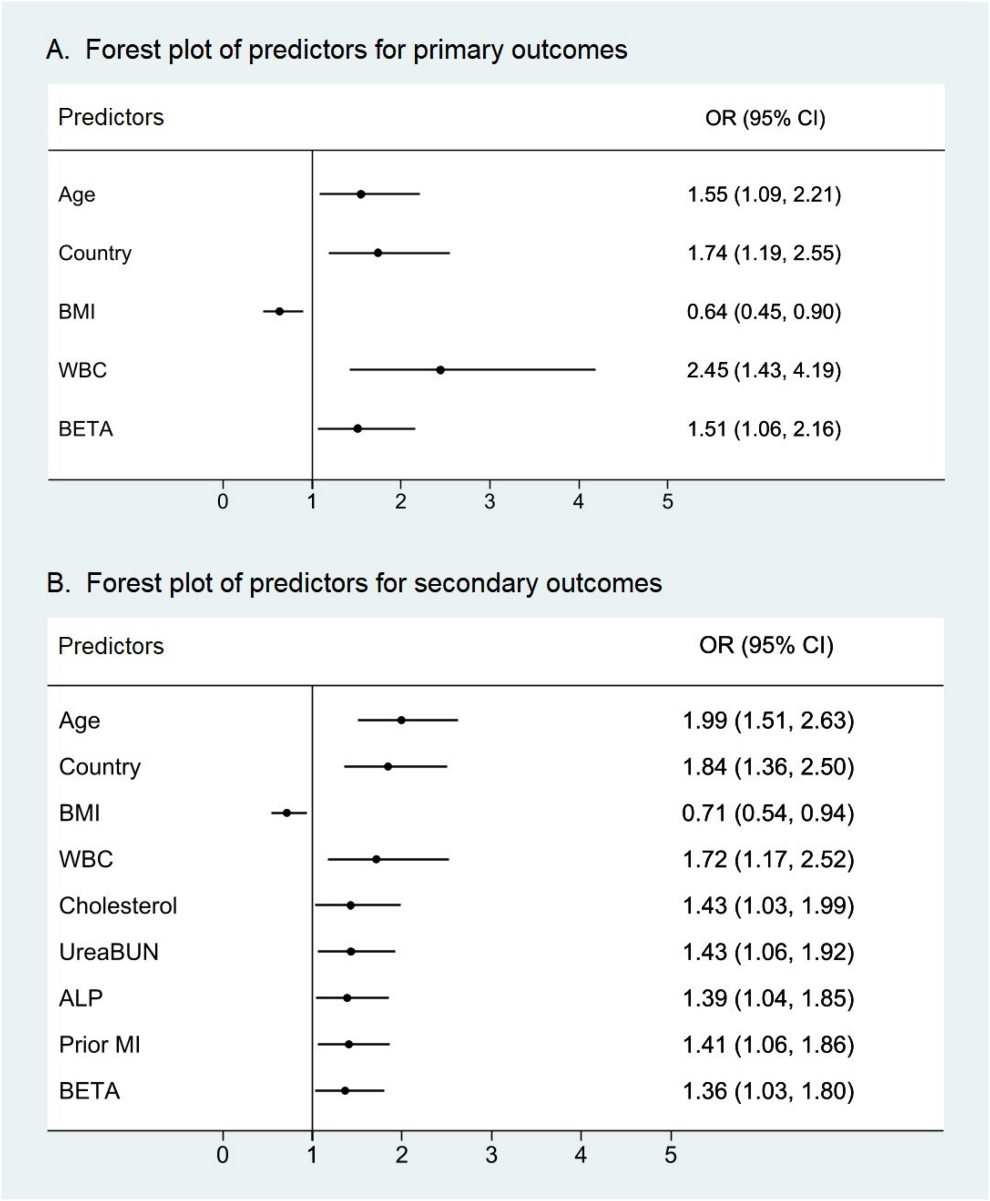
Forest plots of predictors of primary and secondary outcomes with *P* < 0.05. WBC counts were an independent predictor not only for **(A)** the primary outcomes of cardiovascular death, myocardial infarction, and stroke but also for **(B)** the secondary outcomes of all-cause death, cardiovascular death, myocardial infarction, stroke, and hospitalization for heart failure. WBC count displayed the high variable importance measure of all predictors with *P* < 0.05. ALP, alkaline phosphatase; BETA, β-receptor blocker; BMI, body mass index; MI, myocardial infarction; WBC, white blood cell.

The results of multivariate Cox proportional hazards models are shown in Figure 3. In multivariate Cox proportional hazards models for predicting primary outcomes at 4 years, WBC counts were an independent predictor of events (OR = 2.445, 95% CI 1.427–4.190, *P* = 0.001). In addition, age, country, BMI, and β-receptor blockers were also independent predictors of primary outcome events. For the secondary outcomes at 4 years, in Cox proportional hazards, WBC counts were significantly predictive of events with a similar magnitude (OR = 1.716, 95% CI 1.169–2.521, *P* = 0.006). At the same time, age, country, BMI, cholesterol, urea/BUN, ALP, prior MI, β-receptor blockers, and smoking status were also independent predictors of secondary outcome events. It is worth noting that in Asia, the risk of primary and secondary outcomes in patients with stable coronary heart disease in developing countries was 1.74 and 1.84 times higher, respectively, than that in developed countries.

## Discussion

With the aging population, the burden of coronary heart disease in Asia is becoming heavier than in Europe and America. On the other hand, with the development of PCI, the number of patients with stable coronary heart disease is increasing, presenting a huge challenge for its management in Asia. In a secondary analysis of this prospective cohort study of patients with stable coronary heart disease, we explored the association between WBC counts and clinical cardiovascular outcomes in the Asian population. We found that patients with stable coronary heart disease in the lowest WBC quartile had significantly lower incidences of primary and secondary outcome events than patients in other WBC quartiles. After adjusting for the effects of other factors by a multivariable Cox proportional hazards model, we found that WBC count was an independent predictor for primary and secondary outcomes. Therefore, the inclusion of WBC count in the risk assessment system of patients with coronary heart disease has important clinical and public health significance.

Among the general population, the association between WBC count and the risk of coronary heart disease has been confirmed (7, 16). Li et al. (6) conducted a follow-up study on 5,242 Japanese residents aged 40–69 years and found that WBC count was positively associated with the risk of coronary heart disease among the general Japanese population. Karino et al. (17) followed 2,879 men who were free of CHD at baseline for 8 years and found that higher total WBC counts were associated with an increased risk of incident CHD. Akinyelure et al. (8) followed 15,758 participants without a history of coronary heart disease for 11.4 years and found that WBC count was associated with an increased risk of incident CHD. Imano et al. (18) followed 4,492 Japanese men aged 40–59 for 9 years and found that WBC count is a predictor of acute myocardial infarction among Japanese middle-aged men. While all the above studies were conducted in the general population, this study focused on patients with stable coronary heart disease. Theoretically, our study can better reflect the relationship between WBC count and prognosis in patients with stable coronary heart disease. To our knowledge, this is the first multicenter study conducted in an Asian population with stable coronary heart disease that reported the association between WBC counts and cardiovascular events.

The latest research results suggested that the relationship between WBC count and the prognosis of patients with stable coronary heart disease might be related to telomere attrition, cytokines, lipid metabolism, abnormal aggregation, and so on. Recent studies have shown that WBC telomere attrition is associated with the onset of coronary heart disease (19–22), and the WBC count is, to some extent, associated with WBC telomere attrition. Higher WBC counts are associated with faster proliferation and differentiation of WBCs, leading to shorter telomeres. WBC release cytokines that further recruit macrophages and promote the proliferation of smooth muscle cells within the blood vessel walls. In addition, protease secretion leads to endothelial damage in coronary vessels, exposing thrombogenic collagen and predisposing the vessels to thrombus formation (23). The association patterns between peripheral WBC counts and serum lipid levels differ by sex, age, lipid profile, and leukocyte subset (24). They might contribute differently to atherosclerosis. For example, triglycerides and HDL cholesterol may be directly involved in leukogenesis, although the precise mechanism of this relationship and direction of causation are currently ill-defined (25). For the abnormal aggregation of WBCs, the formation of platelet–leukocyte aggregates has been reported in patients with coronary heart disease (26), suggesting that high WBC counts may be one of the possible mechanisms of disease progression.

Our study differs considerably: in analyzing patients with stable coronary heart disease—many of whom have WBC counts within the normal range—we hypothesized that WBC counts would provide useful prognostic information. In essence, this analysis addresses a commonly asked question: Can WBC counts play a role in assessing the risk of cardiovascular events in patients with stable coronary heart disease? Patients with high WBC counts represent an important group that deserves special focus, as they may be at a higher risk of cardiovascular events. Among the 1,933 study participants enrolled in the STABILITY trial with stable coronary heart disease, we found that cardiovascular events were 1.72 times more common in participants with WBC counts ≥5.3 GI/L than in participants with WBC counts <5.3 GI/L, including all-cause death, cardiovascular death, myocardial infarction, and major complications such as stroke and hospitalization for heart failure. In addition, we found that patients with stable coronary heart disease in developed countries (Korea and Japan) had a lower risk of cardiovascular events than those in developing countries (China, Thailand, and the Philippines). This was similar to previous research (27). Our data suggest that developing countries should remain a key focus for the management of patients with stable coronary heart disease in the future.

Patients with stable coronary heart disease have a high incidence of cardiovascular events, and the occurrence of cardiovascular events may be challenging to assess and manage accurately (28). In addition to standard clinical assessment, stress testing, coronary angiography, or coronary CTA are often used for risk stratification in patients with stable coronary heart disease (29–31). While useful, these methods have limitations, including cost and availability. A widely available, inexpensive, and easily interpreted tool, such as a blood test, would be an attractive option to support clinical judgment in assessing the risk of cardiovascular events in patients with stable coronary heart disease. The WBC count can be obtained in almost any medical institution, making it a convenient, fast, and low-cost tool. Although there appears to be an association between higher WBC counts and increased cardiovascular event risk, it remains unclear how individual results could be interpreted at a clinical level. Evaluating the cost-effectiveness of a WBC-leveraged clinical approach, including the development of concentration-specific counts for interpretation, is worth exploring.

### Study strengths and limitations

The strengths of our study lie in the fact that the STABILITY trial was a large, prospective, randomized trial conducted across many countries. It involved not only a homogeneous cohort of patients with stable coronary heart disease but also had high-quality, long-term follow-up with centrally adjudicated endpoints. These factors made our analysis more credible. However, there are some limitations. Patients received a high standard of modern secondary prevention protocols during follow-up, including medication, smoking cessation, and advice on weight loss and regular physical activity. Although multivariate adjustments were made in the analysis, unmeasured confounding factors could not be completely excluded. For example, infection causes a temporary increase in WBC counts (32), which may have unknown effects on the results. In our study, it was unclear whether participants with higher WBC had a hematologic emergency. This also introduced a potential bias in our study. Our analysis included only Asian populations, and only 21.11% of the study population was female, so whether our results can be extrapolated to other populations is still worthy of further study. Integrating WBC counts with other variables and performing a holistic analysis to provide a probability of cardiovascular events may be a future approach that could lead to a better assessment of patients with stable coronary heart disease. There is clearly a need for more data in this area.

## Conclusions

In conclusion, we analyzed data from the Asian population in the STABILITY trial and found differences in primary and secondary outcomes among patients with stable coronary heart disease based on their different WBC counts. In the first quartile, the probability of MACEs in patients with WBC ≥5.3 GI/L was 2.45 times higher than in those with WBC <5.3 GI/L. Analogously, the probability of the composite outcome of all-cause death, cardiovascular death, myocardial infarction, stroke, and hospitalization for heart failure was 1.72 times. WBC count may be an independent predictor of cardiovascular events in patients with stable coronary heart disease. This finding may help in managing the large number of patients with stable coronary heart disease by identifying potentially high-risk groups. It is reasonable to hypothesize that patients with stable coronary heart disease with relatively higher WBC counts could benefit from more aggressive diagnostic or therapeutic management. Implementation of clinical guidelines may consider WBC count as an important reference for the management of patients with stable coronary heart disease. For higher WBC patients, the following suggestions should be considered, avoidance of tobacco use, exercise prescription, and more aggressive medical management of risk factors and so on. WBC count may provide a new idea to inform the most cost-effective management approaches for this important group of patients.

## Data Availability

The datasets presented in this study can be found in online repositories. The names of the repository/repositories and accession number(s) can be found from the following: Unique identifier: NCT00799903; https://clinicaltrials.gov/ct2/show/NCT00799903.
